# 1-Hydroxypyrene–A Biochemical Marker for PAH Pollution Assessment of Aquatic Ecosystem

**DOI:** 10.3390/s100100203

**Published:** 2009-12-28

**Authors:** Jana Blahova, Kamila Kruzikova, Barbora Kasiková, Pavel Stierand, Jana Jurcikova, Tomas Ocelka, Zdenka Svobodova

**Affiliations:** 1 Department of Veterinary Public Health and Toxicology, Faculty of Veterinary Hygiene and Ecology, University of Veterinary and Pharmaceutical Sciences, Palackeho 1-3, 612 42, Brno, Czech Republic; E-Mails: kruzikovak@vfu.cz (K.K.); svobodovaz@vfu.cz.(Z.S.); 2 Institute of Public Health Ostrava, Partyzanske namesti 7, 702 00 Ostrava, Czech Republic; E-Mails: barbora.kasikova@zuova.cz (B.K.); jana.jurcikova@zuova.cz (J.J.); tomas.ocelka@zuova.cz (T.O.); 3 Czech Hydrometeorological Institute, Kroftova 43, 616 67, Brno, Czech Republic; E-Mail: pavel.stierand@chmi.cz (P.S.)

**Keywords:** aquatic pollution, fish, bile, SPMD, the Svitava and Svratka rivers, polycyclic aromatic hydrocarbons

## Abstract

The aim of the present study was to assess aquatic contamination by polycyclic aromatic hydrocarbons (PAH), using the 1-hydroxypyrene (1-OHP) content in fish bile as a biochemical marker. A total of 71 chub (*Leuciscus cephalus* L.) were collected from seven locations on the Svitava and Svratka rivers in and around the industrial city of Brno, Czech Republic. The levels of 1-OHP were determined by reverse phase HPLC with fluorescence detection after deconjugation. Normalising the molar concentration of the biliary 1-OHP to the biliary protein content reduced sample variation. The content of 1-OHP was correlated with the PAH level in bottom sediment and semi-permeable membrane devices (SPMD), which was analyzed by a combination of HPLC/FLD and GC/MS methods. The highest mean values of 1-OHP were found in fish caught at the Svratka River at locations Modřice (169.2 ± 99.7 ng·mg^−1^ protein) and Rajhradice (152.2 ± 79.7 ng·mg^−1^ protein), which are located downstream from Brno. These values were significantly (*P* < 0.05) higher than those obtained from localities Kníničky (98.4 ± 66.1 ng·mg^−1^ protein) and Bílovice nad Svitavou (64.1 ± 31.4 ng·mg^−1^ protein). The lowest contents of PAH in sediment and SPMD were found at location Kníničky (1.5 mg·kg^−1^ dry mass and 19.4 ng·L^−1^, respectively). The highest contents of PAH in sediment and SPMD were found in Rajhradice (26.0 mg·kg^−1^ dry mass) and Svitava before junction (65.4 ng·L^−1^), respectively. A Spearman correlation test was applied to determine the relationship between biliary 1-OHP and the sum of PAH in sediment and SPMD. A positive, but no statistically significant correlation was found. The main impact sources of elevated level of PAHs in sites located downstream from Brno are most probably intensive industrial and agricultural activities and domestic waste.

## 1. Introduction

Polycyclic aromatic hydrocarbons (PAH) are a class of hydrophobic environmental organic pollutants that are widely distributed in the environment. PAH are formed as a result of the incomplete burning of coal, gas, wood, garbage, or other organic substances, such as tobacco and charboiled meat. PAH generally occur as complex mixtures, not as single compounds [1,2]. Some of these compounds are environmentally important because they are, or can become, carcinogenic or mutagenic. The US Environmental Protection Agency has identified 16 PAH as particularly significant due to their toxicity to aquatic organisms and mammals [3]. The 16 priority pollutant PAH are: acenaphthylene, acenaphthene, anthracene, benzo[*a*]anthracene, benzo[*a*]pyrene, benzo[*b*]fluoranthene, benzo[*k*]-fluoranthene, benzo[*ghi*]perylene, chrysene, dibenzo[*ah*]anthracene, fluoranthene, fluorene, indeno[*1,2,3-cd*]pyrene, naphthalene, phenanthrene and pyrene. These compounds have 2–6 fused rings and molecular weights of 128–278 g·mol^−1^ [1].

PAH infusion into the aquatic environment are primarily from two sources: (a) the movement of water containing dissolved and particulate constituents derived from watersheds; (b) atmospheric deposition both from precipitation and dry deposition from airsheds. The major sources of PAH in the aquatic environment include urban runoff, wasterwater effluents, industrial outfalls, atmospheric deposition, and spills and leaks during the transport and production of fossil fuels [1]. As a consequence of their hydrophobic characteristics, PAH tend to concentrate in sediments where they may be present at levels thousands of times higher than those in the overlying water. Fish living in PAH contaminated environments absorb these compounds through their body surface and gills or by ingesting contaminated sediment or food [1,4,5].

PAH in the aquatic environment can be transformed by chemical (photo) oxidation or biological transformation. PAH biotransformation occurs in many aquatic organisms, but it is most effective in the liver of fish. PAH are easily metabolized by the phase I enzymes of mixed function oxygenase system to more hydrophilic products (hydroxylated derivates). Some PAH can be excreted directly as unconjugated polar metabolites, but most of them are in the second phase conjugated with sulfate or glucuronic acid, incorporated into the bile, and finally deposited in the gall bladder for elimination from the organism. Therefore, hydroxylated metabolites can be analyzed in bile and can be used as biomarkers for exposure to PAH in fish [6,7]. Many laboratory studies have demonstrated that the presence of biliary PAH metabolites is well correlated with the levels of exposure [8,9], and this trend has been corroborated in a number of field studies [10,11]. Analysis of PAH metabolites is effectively and frequently used in the biological monitoring of human exposure to PAH, especially in the working environment [12,13].

The analysis of bile metabolites is a convenient and relatively rapid method for monitoring PAH contamination in fish [14]. Various analytical techniques have been used to determine metabolite level in bile. Synchronous fluorescence scan [15] or fixed wavelength fluorescence [16] can be used for PAH exposure determination. More detailed information about the metabolite pattern is provided by using chromatography methods. Identification and quantification of individual PAH metabolites requires a more labor-intensive sample preparation and analysis method, and also the availability of pure calibration standards. A limited number of PAH metabolites are commercially available and the majority of these are non-conjugated metabolites. Therefore, sample preparation usually includes an enzymatic treatment for producing free hydroxy PAH. Separation by HPLC [11,16–18] or GC [17] prior to fluorescence or mass spectrometry detection are the more frequently used methods for the identification and quantification of individual metabolites [17].

**Figure 1.**
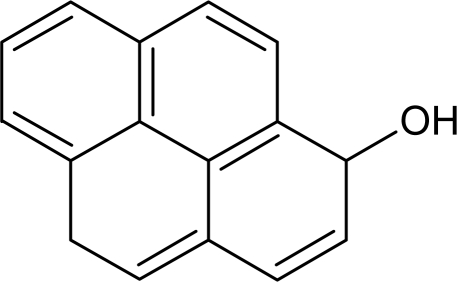
Formula of 1-hydroxypyrene (C_16_H_10_O).

The main metabolite in fish bile is 1-hydroxpyrene (1-OHP), which contributes up to 76% of the sum of PAH metabolites [19]. Other metabolites, detected in considerably lower levels in fish bile, are 1-hydroxyphenanthrene, 1-hydroxychrysene and three metabolites of benzo(*a*)pyrene [5,14]. 1-Hydroxypyrene (Figure 1) is the predominant biotransformation product of pyrene, a widespread and common PAH that is generated by many pyrolytic and petrogenic industrial processes [11,18]. Urinary 1-OHP has been used frequently as a biomarker of human exposure to PAH, especially for worker in oil refineries, tar plants, aluminium plants and coal-burning facilities [20].

Accumulation of PAH metabolites in the gall bladder depend on the level of exposure in environment, but their concentration in the bile is influenced by dietary status. In fish that have not recently fed, metabolites tend to become concentrated in the bile due to resorbtion of water and other low molecular weight compounds through the gall bladder [5]. Vuorinen *et al.* [21] described the possibility of using biliverdin or protein content for the normalization of 1-OHP contents.

The aim of the present study is to assess the contamination caused by PAH in seven selected locations on the Svitava and Svratka rivers in and around the industrial city of Brno (Czech Republic) using 1-OHP content in fish bile as a biomarker. Two locations are situated upstream of the Brno agglomeration and other locations were downstream of the city, where the pollution might be attributed to the intensive anthropogenic activities involving PAH emissions. The content of 1-OHP concentration was correlated with PAH content in sediment samples.

## 2. Material and Methods

### 2.1. Sampling Sites

**Figure 2.**
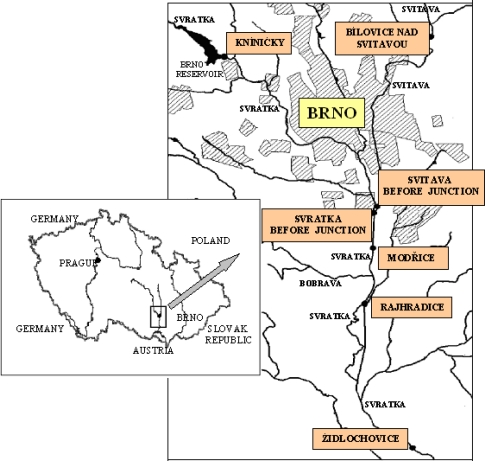
Study areas on the Svitava and Svratka rivers (Czech Republic).

Monitoring was perfomed at seven locations on the Svitava and Svratka rivers in the vicinity of Brno. The sampling sites are shown in Figure 2. Brno, the second-largest city in the Czech Republic, with approximately 370,000 inhabitants, is located in the southeastern part of the country, at the confluence of the Svitava and Svratka rivers. Brno is an important industrial city with highly developed engineering, chemical, textile and food-processing industries. Domestic waste, sewage and other effluents from industrial sources are most likely responsible for the PAH input into the area’s aquatic ecosystem.

The locations studied on the Svitava River are Bílovice, upstream from Brno (*Bílovice and Svitavou*), and a second site downstream from Brno but upstream from the confluence with the Svratka River (*Svitava before junction*). The sites on the Svratka River were: a location upstream from Brno (*Kníničky*), a site downstream from Brno before the confluence with the Svitava River (*Svratka before junction*), plus *Modřice*, *Rajhradice*, and *Židlochovice*, all downstream from the confluence of the two rivers.

### 2.2. Animals and Sampling

**Figure 3.**
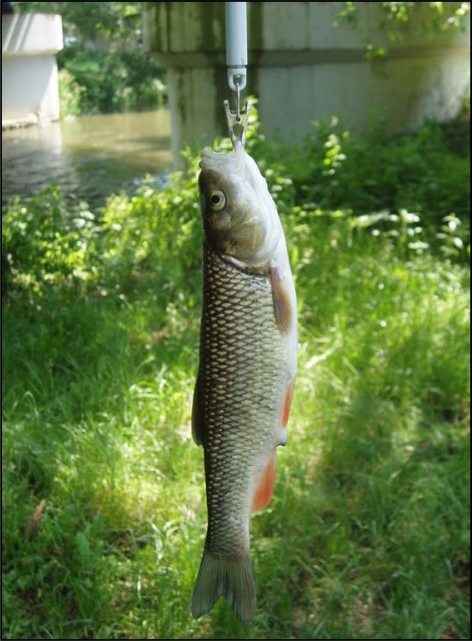
The indicator species-chub (*Leuciscus cephalus* L.).

Chub (*Leuciscus cephalus* L., Figure 3) was selected as the most suitable indicator species, due to its relative abundance at the sampling sites as well as being sensitive bioindicators of freshwater pollution [22,23]. At each location, seven to 14 male chub were caught by electrofishing in June, July and September 2008. A total of 71 specimens were analyzed. Body weight was measured and scales were collected for age determination. Sex was determined macroscopically. The biometric characteristics of fish are given in Table 1. Bile was drawn by hypodermic needle through the exposed gall bladder and emptied into an Eppendorf tube. Samples were immersed in dry ice and taken to the laboratory, where they were stored at −80 °C until analysis.

At each location, composite bottom sediment was collected into dark glass bottles and stored at −18 °C. The collection of sediment samples was taken from an area about 5 m^2^ as a surface sampling from a depth of about 5 cm. The pooled sample was obtained from four spots in each locality. Sediment samplings were taken three times a year, in February, May and October 2008.

**Figure 4.**
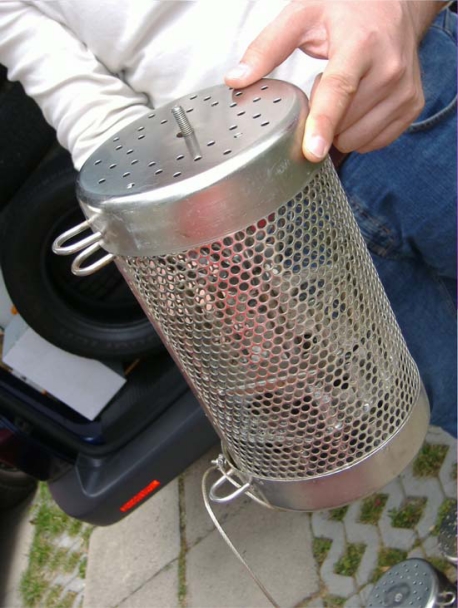
Semi-permeable membrane device.

One passive sampler (Figure 4), a semi-permeable membrane device (SPMD), was placed at each site for one month in spring (May 7–June 4) and autumn (September 17–October 15). Semi-permeable membrane device sampler consists of a horizontal thin-walled tub from nonporous low density polyethylene, filled with synthetic lipid (triolein). The SPMD were commercially manufactured (Exposmeter AB, Sweden). After exposure the sampler membranes were rinsed briefly with distilled water to remove superficial deposits and placed on ice for transport to the laboratory. They were stored in the laboratory at −18 °C until analysis.

### 2.3. Determination of 1-Hydroxypyrene in Bile

1-Hydroxypyrene occurring in sampled fish bile was determined by the reversed phase HPLC/FLD method described in detail earlier by Blahova *et al.* and Hosnedl *et al.* [11,18]. Bile samples were deconjugated with an enzyme mixture of glucuronidase and arylsulphatase and were purified on a LiChrolut^®^EN column (Merck). Purified bile hydrolysates were analyzed with HPLC system consisting of Alliance HPLC system, 2475 fluorescence detector (λ_ex_ = 364 nm, λ_em_ = 384 nm) and Empower data system (Waters, USA). The analytical column was Polaris C18–A, 150 × 4.6 mm I.D. with 3μ packing, thermostatted at 35 °C (Varian Inc.). Separation was performed in 12 min using an acetonitrile:water mobile phase. The linear gradient was as follows: t = 0 min: 65% acetonitrile, t = 5 min: 70% acetonitrile, t = 10 min: 80% acetonitrile, t = 12 min: 65% acetonitrile. Recovery and reproduction of the analyte were improved by the addition of ascorbic acid (1 mg·mL^−1^) to the water eluent. Each sample was hydrolysed and subjected to HPLC analysis twice. Performance characteristics of applied method obtained through the validation procedure were as follows: the limits of detection: 0.118 ng.ml^−1^, limit of quantification: 0.392 ng.ml^−1^, recovery: 90.1 ± 3.9% and repeatability (as relative standard deviation): 6.3%. 1-Hydroxypyrene concentration was normalised to the bile protein content. Total biliary protein was measured by a modified spectrophotometric method (570 nm) of Smith *et al.* [24], using bicinchoninic acid and bovine serum albumin.

### 2.4. Determination of PAH in Sediment and SPMD Samples

The analysis of phenanthrene, anthracene, fluoranthene, pyrene, benzo[*a*]anthracene, chrysene, benzo[*b*]fluoranthene, benzo[*k*]fluoranthene, benzo[*a*]pyrene, dibenzo[*ah*]anthracene, benzo[*ghi*]-perylene and indeno[*1,2,3-cd*]pyrene was performed by combination of HPLC/FLD and GC/MS methods. Due to low selectivity of fluorescence detector for two and three ring PAH, GC/MS method was used for identification and quantification of low MW PAH.

Before chromatography analyses, sediment samples were lyophilized and homogenized, and then were extracted using microwave accelerated extraction. The membranes were dialysed by hexan and dialysates were used for PAH analysis. The HPLC/FLD analysis was carried out by HPLC system from Agilent (HP 1100 series) using a reversed phase column (Lichrospher PAH 250 × 3 mm) and water/methanol gradient for separation at 30 °C. *ChemStatiton software* was used for data processing. Gas chromatography mass spectrometric analysis was performed on a ThermoFinnigan TRACE DSQ™ Single Quadrupole GC/MS with a 30 m DB-5MS capillary column (0.25 mm I.D. × 0.25 μm film) and operated in electron ionization mode. A commercial GC/MS software *Xcalibur 1.4 version* was used for data processing. Determination of PAH in sediment and SPMD samples was carried out by laboratories of the Centre of Hygienic Laboratories Ostrava, which are accredited by the Czech Accreditation Institute.

### 2.5. Statistical Analysis

Statistical analysis of the data was performed using the program *Statistica 8.0 for Windows* (StatSoft, Inc. USA). Values of 1-OHP in fish bile were tested for normal distribution using Kolmogorov–Smirnov test and data were log-transformed to improve the homogeneity of variance. A one-way analysis of variance (ANOVA) was applied to the differences in 1-OHP content between sampling locations. Individual differences between the means were tested successively using LSD test (Fischer Least Significant Difference method) and *P* < 0.05 was chosen as the level of significance. The relationship between biliary 1-OHP and PAH content in sediment was assessed using Spearman′s correlation coefficient.

## 3. Results

**Figure 5.**
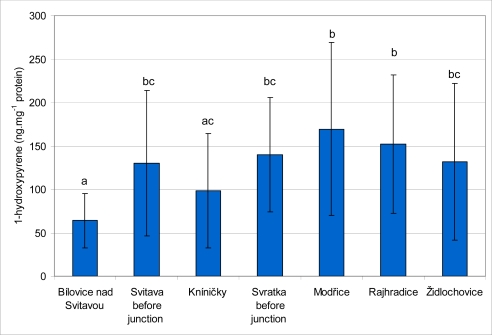
Content (mean ± standard deviation) of 1-hydroxypyrene in chub bile samples at monitored locations. Significant differences (*P* < 0.05) are indicated by alphabetic superscript.

The results of 1-OHP content in fish bile are summarized in Figure 5. The highest mean values of 1-OHP were found in fish caught at the Svratka River at localities Modřice (169.2 ± 99.7 ng·mg^−1^ protein) and Rajhradice (152.2 ± 79.7 ng·mg^−1^ protein). These values were significantly (*P* < 0.05) higher than those obtained from the Kníničky (98.4 ± 66.1 ng·mg^−1^ protein) and Bílovice nad Svitavou (64.1 ± 31.4 ng·mg^−1^ protein) sites. There were no significant differences between the sites at Modřice, Rajhradice, Svratka before junction (139.9 ± 66.2 ng·mg^−1^ protein), Židlochovice (131.2 ± 90.2 ng·mg^−1^ protein) and Svitava before junction (130.4 ± 83.8 ng·mg^−1^ protein). The lowest mean value of 1-OHP content was found at Bílovice nad Svitavou, and this value was significantly lower than those obtained at all other sites with the exception of Kníničky. The lowest level of 1-OHP from all of the individual samples was found at Kníničky (15.3 ng·mg^−1^ protein), and the highest level was observed at the Rajhradice site (350.4 ng·mg^−1^ protein).

**Figure 6.**
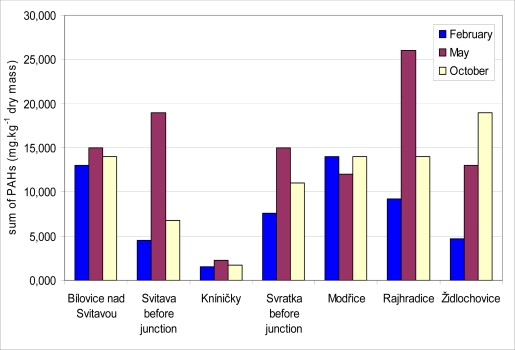
PAH content (in mg·kg^−1^ dry mass) in sediment samples at monitored locations.

**Figure 7.**
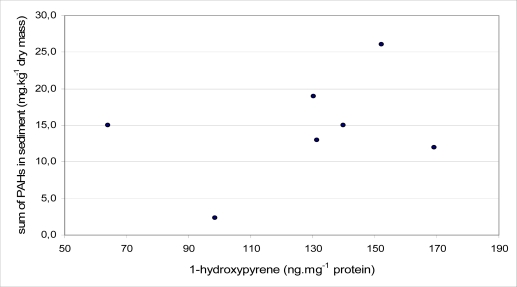
Correlation between content of biliary 1-OHP and PAH in sediment in May (*r* = 0.68, *P* > 0.05).

In all locations, sediment samples were collected three times a year (in February, May and October 2008) for PAH determination. Results of total PAH levels in sediment samples are presented in Figure 6. PAH levels in sediment ranged between 1.5 and 26.0 mg·kg^−1^ dry mass in all localities during the year. The lowest content of PAH was obtained in locality Kníničky in February. In this locality was the PAH content the lowest in case of all sampling (2.3 mg·kg^−1^ dry mass in May and 1.8 mg·kg^−1^ dry mass in October). On the other hand the highest content of PAH was obtained in locality Rajhradice in May (26.0 mg·kg^−1^ dry mass). A Spearman correlation test was applied to find the relationship between biliary 1-OHP and total PAH in sediment for different months. A positive correlation was found in each month, but there were no statistically significant differences. The correlation coefficients attributed to the sediment samples collected in February, May and October were 0.32, 0.68 and 0.19, respectively. The highest correlation coefficient between the content of biliary 1-OHP and PAH in the sediment samples was found in May. This correlation is presented in Figure 7.

**Figure 8.**
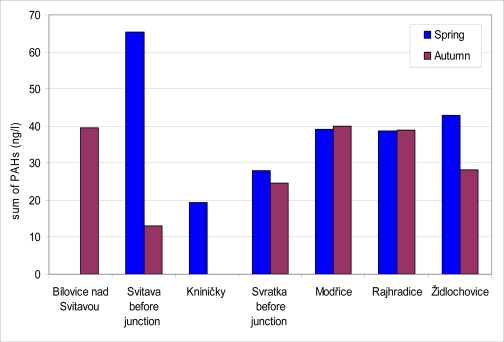
PAH content (in ng·L^−1^) in SPMD at monitored locations.

Amounts of PAH sequestered in the SPMD from water are given in Figure 8. Because of technical problems, SPMD from Bílovice nad Svitavou (in spring) and from Kníničky (in autumn) did not produce samples. PAH levels in SPMD ranged between 13.0 and 65.4 ng·L^−1^ in all localities during the year. The content of PAH did not differ during the year in locations monitored with exception of the locality Svitava before junction. In this locality PAH content was the highest (65.4 ng·L^−1^) in all spring samplings, on the other hand the lowest PAH content (13.0 ng·L^−1^) was found here in all autumn samplings. By a correlation analysis we confirmed a non-significant positive correlation between biliary 1-OHP and PAH contents in SPMD. The correlation coefficients attributed to the samples obtained in spring and autumn were 0.09 and 0.37, respectively.

## 4. Discussion

The monitoring of the Svitava and Svratka rivers was carried out within the framework of the project “New methods for the monitoring of city agglomeration effect at quality parameters of fluvial environments with emphasis on identification of endocrine compounds” (MSM Project No. 2B06093), which started in 2006. This project is focused on the assessment of aquatic pollution by environmental endocrine disruptors at seven selected locations situated upstream and downstream from Brno city using analyses of biochemical markers in fish and pollutant contents in several matrices (fish muscle, sediment and a passive sampler) [25,26]. One group of the important and widespread endocrine modifying compounds is PAH, which are generated mainly by anthropogenic activities [27,28]. Generally, environmental exposure of fish to PAH is assessed by monitoring their environment (sediment, water, *etc.*), and also the contaminants levels in the aquatic environment, which usually involves the determination of parent PAH in sediment [11,18,29] or passive sampler [30]. Furthermore, the biomonitoring of PAH exposure should include determination of PAH metabolites in fish bile. Many authors have shown that 1-OHP is the most common metabolite detected and is regarded as the best general indicator of PAH exposure in fish [11,14,18].

The evaluation of biliary 1-OHP content in chub caught in 2007 in the same localities within the frame of this project is presented in a study by Blahova *et al.* [11]. These results are in accordance with results obtained in 2008, which were further extended by analysis of PAH content in SPMD. The highest concentrations of 1-OHP measured were obtained at the Rajhradice and Modřice sites (the Svratka River), which are situated downstream from Brno. The levels of 1-OHP in these locations were significantly (*P* < 0.05) different only from the Bílovice nad Svitavou and Kníničky sites, which are situated upstream from Brno. The Modřice site is situated below the confluence of Svitava and Svratka rivers and the effluents from the municipal waste water treatment plant (WWTP) below Brno. This WWTP treats wastewater conveyed by a system of sanitary sewers from the city of Brno, and increasingly, from a system of pumping stations that connects other municipalities to the WWTP. The Rajhradice site is located 4 km downstream from the WWTP and the water quality is adversely affected not only by WWTP contamination, but from another source of PAH contamination possibly coming from a tributary, the Bobrava River. The Bobrava River is an important tributary of the Svratka River (2 km upstream from Rajhradice), and the water quality could possibly be affected by industrial activities in nearby cities (e.g., the Rosice Coal Mine) or by the WWTP in Tetčice, Popovice or Radostice. However, the results of biliary 1-OHP obtained at Svratka River in locality Židlochovice (5 km downstream from Rajhradice) indicate that there are probably no further sources of PAH along this river, and similarly, at the Svratka before junction and the Svitava before junction, which is upstream from Modřice, where the levels of 1-OHP are lower. The effect of city agglomeration on PAH in aquatic ecosystem was studied by many authors [7,8,18]. Hosnedl *et al*. [18] monitored the PAH pollution in the vicinity (upstream and downstream) of large urban areas represented by the cities of Prague and Pardubice, which are located on the Vltava and Elbe rivers, respectively (Czech Republic). The highest levels of 1-OHP in fish and PAH content in sediment from Elbe River were obtained in Srnojedy, situated downstream from the industrial city of Pardubice. Similarly, the highest levels of biliary 1-OHP and PAH content from Vltava River were found in Klecany, which is located downstream from the Prague industrial area. Similar results were reported by Barra *et al.* [31], who investigated PAH levels in sediments taken from the middle stretch of the Biobio River in the south central Chile. The highest contents were detected downstream from an industrial discharge. Johnson-Restrepo *et al.* [7] evaluated the PAH pollution in sites located along the Atlantic coast of Columbia. The highest PAH contamination was obtained at Cartagena Bay, a typical estuary located off-shore from the city of Cartagena (population ∼ 895,000). This body of water receives wastewater from the city through a submarine outfall, and industrial inputs from a crude oil refinery, pesticides, and petrochemical plants, among others, in addition to heavy ship traffic. The mean values of PAH metabolites and PAH in sediment were severalfold higher than those obtained in reference sites located to the south and north of Cartagena Bay, respectively.

The lowest concentrations of 1-OHP were observed at locations situated upstream from Brno (Kníničky and Bílovice nad Svitavou). Bílovice nad Svitavou (the Svitava River) was chosen as a control location, which reflects the state of surface water before entering the Brno agglomeration. Kníničky (the Svratka River) is situated below the Brno Reservoir dam and represents a typical area affected by the dam, so it stands to reason that the lowest level of PAH in sediment was obtained in Kníničky. This result was in accordance with results obtained in 2007 within the frame of this project by Blahova *et al.* [11], where the lowest level of PAH in sediment was also found in Kníničky. The fact that there is a much lower content of both indices downstream of the dam indicate that PAH are likely adsorbed onto sediment particles in the reservoir above the dam. Consequently, there is minimal presence of these compounds downstream of the dam. These compounds tend to bind rapidly to organic matter and small particles in the water column as well in sediments in the reservoir above the dam [1,32]. Ruddock *et al.* [14] cited that PAH level in sediment can reach concentrations of 3 or 4 orders of magnitude higher than in the overlying water.

Several studies have indicated a statistically significant relationship between fish bile metabolite and PAH in sediment [11,18]. Hosnedl *et al.* [18] documented a positive correlation (*P* < 0.05) for chub and bream, and the correlation coefficients were 0.81 and 0.76, respectively. A strong positive and significant correlation (*P* < 0.01, *R* = 0.95) was observed between the total concentration of the five PAH in sediment samples (phenanthrene, anthracene, pyrene, benzo[*a*]pyrene and benzo[*k*]-fluoranthene) and 1-hydroxypyrene concentration measured in the bile of marine polychaete (*Nereis diversicolor*) collected on a sand flat at Store Havelse (Roskilde Fjord, Denmark) [29]. In this present study there was a positive correlation between 1-OHP and PAH in sediment as well, but there was no statistical significance. The highest correlation coefficient (*R* = 0.68) was obtained in the case of sediment samples collected in May. No significant correlation was caused by them, and the sampling of sediment and fish bile was not performed at the same time. Because of a technical problem, the part of bile samples from locality Modřice was sampled as late as September.

Semi-permeable membrane device techniques combined with biomarkers may provide useful information on bioavailability and toxicological impact of hydrophobic compounds on the aquatic environment [33]. Positive correlation between uptake of pyrene in SPMD and its metabolites in fish bile was shown by Verweij *et al.* [31]. Harman *et al.* [28] investigated accumulation of PAH by SPMD. They exposed atlantic cod (*Gadus morhua*) and SPMD over 4 weeks to a mixture simulating the composition of PAH in water discharge in the North Sea. Regression analysis of accumulations of individual compounds in fish and SPMD showed reasonable but compound-specific correlation, the correlation coefficients ranged from 0.54 to 0.85. In our study we did not obtain significant correlation between biliary 1-OHP and PAH content in SPMD which we attribute to different sampling times.

## 5. Conclusions

The results presented in our study indicate the highest PAH contamination of sites situated downstream from Brno (especially at Rajhradice and Modřice), where the pollution might be predicated by intensive anthropogenic activities. The main impact sources of the elevated PAH level are the most probably intensive industrial and agricultural activities as well as domestic waste and sewage. Although we did obtain a positive but not a statistically significant correlation between the levels of biliary 1-hydroxypyrene and PAH content in sediment and SPMD, on the basis of our results and those of other studies it is evident that this metabolite is a suitable biochemical marker for the assessment of aquatic pollution caused by PAH.

## Figures and Tables

**Table 1.** Characteristics of male chub (*Leuciscus cephalus* L.) from sampling sites.

**Locality** (river km)	**Fish** n	**Age** years (min–max)	**Weight** ± **SD** g
	*Svitava River*		

Bílovice nad Svitavou (18.0)	10	4.2 (3.5–5.5)	141 ± 30
Svitava before junction (0.6)	7	3.1 (2.5–3.5)	168 ± 56

	*Svratka River*		

Kníničky (56.2)	10	3.9 (2.5–5.5)	274 ± 103
Svratka before junction (40.9)	13	4.1 (2.5–5.5)	284 ± 170
Modřice (38.7)	7	4.2 (3.5–5.5)	293 ± 132
Rajhradice (35.0)	14	3.8 (2.5–4.5)	298 ± 151
Židlochovice (30.0)	10	3.5 (2.5–4.5)	222 ± 101
